# Sex-Specific Mechanisms of Central Sensitization: A Narrative Review of the Neuroimmune Architecture of Chronic Pain

**DOI:** 10.7759/cureus.111510

**Published:** 2026-06-25

**Authors:** Diogo Andrade Pereira

**Affiliations:** 1 Physical Medicine and Rehabilitation, Hospital Beatriz Ângelo, Lisbon, PRT

**Keywords:** central sensitization, epigenetic regulation, gonadal hormones, kcc2, neuroimmunology, neuropathic pain, precision medicine, sex characteristics, spinal microglia, t lymphocytes

## Abstract

Chronic pain disproportionately affects women, yet analgesic development has been built predominantly on male biological substrates, reflecting a now-refuted assumption of biological symmetry that excluded female subjects from preclinical investigation. Central sensitization, the pathological amplification of central nervous system signalling that produces pain hypersensitivity, is increasingly recognized as a sex-dependent neuroimmune phenomenon with distinct cellular and molecular signatures in males and females. This narrative review, prepared in accordance with the Scale for the Assessment of Narrative Review Articles (SANRA), synthesizes evidence challenging the "one-size-fits-all" paradigm and proposes a transition toward biological-sex-informed precision analgesia. In males, central sensitization is predominantly mediated by spinal microglial activation via the P2X4 receptor-brain-derived neurotrophic factor (BDNF) axis, culminating in potassium-chloride cotransporter type 2 (KCC2) downregulation and GABAergic disinhibition. In females, it proceeds through spinal T-lymphocyte infiltration and direct pro-excitatory actions of interferon-γ and tumor necrosis factor-α on dorsal horn neurons, a pathway largely refractory to microglial-targeted intervention. Gonadal steroids act as neuroimmune modulators: 17β-estradiol amplifies central excitability, whereas testosterone is antinociceptive. Epigenetic mechanisms consolidate these signals into durable transcriptional programs that sustain sensitization after injury resolves. The failure of microglial-targeted therapies in female-predominant cohorts reflects a sex-mismatched strategy. Inclusion of biological sex as a primary variable in pain research and practice is both a scientific and ethical imperative.

## Introduction and background

The paradigm of symmetry and male bias in pain science

Chronic pain medicine stands at an inflection point. Despite a continuously expanding therapeutic arsenal-encompassing high-frequency spinal cord stimulation, dorsal root ganglion neuromodulation, and precision-guided pharmacological blockade, one epidemiological reality has proven stubbornly resistant to intervention: the global burden of chronic pain falls disproportionately on women. Population-based data consistently show that females exhibit higher prevalence, greater severity, and longer duration across most major chronic pain syndromes, including fibromyalgia, migraine, temporomandibular disorders, irritable bowel syndrome, and chronic widespread pain [1-4]. Yet for much of the twentieth century, the foundational science underpinning analgesic development proceeded as if biology were indifferent to sex. The central thesis of this review is that the neuroimmune machinery sustaining central sensitization (CS) is sexually dimorphic at the cellular level, with direct consequences for analgesic strategy.

The institutional male bias in preclinical pain research, the systematic overrepresentation of male subjects, was not a passive omission. It reflected the now-refuted assumption that estrous-cycle hormonal fluctuations would introduce unacceptable variability into female rodent data [1]. The practical consequence has been a durable translational barrier: an analgesic pipeline derived predominantly from male biological substrates and deployed in clinical populations in which the majority of patients are female [5,6]. Recognizing biological sex not as a confounding variable but as a primary, mechanistically determinative biological variable represents a fundamental conceptual shift in contemporary pain science.

CS: the molecular substrate of pain chronicity

At the neurobiological core of chronic pain lies CS, an amplification of neural signalling within the CNS that translates transient nociceptive input into sustained pain hypersensitivity [7,8]. As defined by Woolf, CS describes a state in which the somatosensory nervous system operates in a mode of pathological gain: normally innocuous stimuli elicit pain (allodynia) and noxious inputs are exacerbated (hyperalgesia) [7]. It represents a maladaptive reprogramming of nociceptive circuits that persists independently of the original peripheral trigger.

The architecture of CS rests on three interdependent pillars: (i) excitatory synapses become stronger and more responsive (through N-methyl-D-aspartate (NMDA) and α-amino-3-hydroxy-5-methyl-4-isoxazolepropionic acid (AMPA) receptor activation); (ii) inhibitory control collapses (gamma-aminobutyric acid (GABA)-ergic and glycinergic disinhibition); and (iii) neuroinflammatory cascades sustain the resulting neuronal hyperexcitability [7,9]. Crucially, the cellular identity, relative contribution, and molecular mechanism of each pillar differ between males and females, reframing CS not as a singular pathophysiological entity, but as a family of mechanistically distinct neuroimmune syndromes converging on a shared clinical phenotype.

The neuroimmune dichotomy as a pathophysiological landmark

The central thesis of this review is that the neuroimmune machinery sustaining CS is sexually dimorphic at the cellular level. In males, the transition from acute to chronic pain is driven largely by spinal immune cells (microglia). This process operates through a specific molecular cascade, the P2X4 receptor-brain-derived neurotrophic factor (BDNF)-potassium-chloride cotransporter type 2 (KCC2) axis, which, when pharmacologically interrupted, reverses neuropathic allodynia in male but not female animals [9-11]. In females, this microglial program is largely bypassed: the adaptive immune system, through T-lymphocyte infiltration of the spinal cord parenchyma and release of pro-excitatory cytokines, assumes the mechanistic role that microglia occupy in males [6,11,12]. This is not a quantitative difference in the magnitude of neuroinflammation; it is a qualitative divergence in the identity of the effector cells, a distinction with substantial therapeutic implications.

Gonadal hormones and epigenetic gatekeeping

Superimposed on this cellular dichotomy is a hormonal regulatory layer of comparable mechanistic importance. Sex steroids operate as dynamic neuroimmune modulators, continuously calibrating synaptic excitability, ion channel expression, and glial reactivity through rapid nongenomic and slower transcriptional mechanisms [13]. 17β-estradiol acts broadly to amplify pain signalling, enhancing excitatory neurotransmission, increasing the expression of pain-related sodium channels, and heightening immune-cell responsiveness, whereas testosterone is largely protective, dampening microglial activation and reinforcing the body's descending pain-inhibitory pathways [14,15]. At the deepest level of biological persistence, epigenetic mechanisms-DNA methylation, histone modification, and noncoding RNA networks-consolidate these hormonal and immune signals into durable transcriptional programs that sustain CS after the initiating injury has resolved [16,17].

Objectives and scope

Despite converging experimental evidence, clinical pain guidelines and interventional protocols remain substantively sex-blind. This narrative review aims to (i) characterize the neuroimmune dichotomy between the microglial P2X4-BDNF-KCC2 axis in males and the T-cell/cytokine-mediated pathway in females [10,11], (ii) evaluate hormonal modulation of nociceptive excitability through 17β-estradiol and testosterone [13,15], (iii) integrate epigenetic mechanisms as molecular substrates for pain chronicity and emerging therapeutic targets [16,17], (iv) analyze the translational gap underlying the failure of glial-targeted therapies in female-predominant clinical cohorts [6], and (v) propose an actionable framework for sex-stratified precision analgesia [18,19].

## Review

Materials and methods

This article is a narrative review, prepared and reported in accordance with the Scale for the Assessment of Narrative Review Articles (SANRA), which emphasizes clarity of rationale, explicit aims, a justified literature search, appropriate referencing, scientific reasoning, and meaningful presentation of evidence [20]. A structured but nonsystematic literature search was conducted across three electronic databases, PubMed/MEDLINE, Scopus, and Web of Science, covering January 2005 to April 2026. This window was selected to encompass the seminal mechanistic discoveries underpinning the neuroimmune dichotomy [9,10] together with the most recent clinical-translational advances [6,19,21]. The search combined Medical Subject Headings (MeSH) terms and free-text keywords, including “sex differences,” “biological sex,” “central sensitization,” “spinal microglia,” “P2X4 receptor,” “T lymphocytes,” “BDNF,” “KCC2,” “17β-estradiol,” “testosterone,” “neuroimmune interaction,” “epigenetics,” and “precision analgesia.” Boolean operators (AND, OR) were applied to refine intersections between biological sex and the molecular mechanisms of pain chronification. As an illustrative example, the PubMed/MEDLINE search combined the following: ("sex differences" OR "biological sex") AND ("central sensitization" OR "neuropathic pain") AND ("microglia" OR "P2X4" OR "T-lymphocytes" OR "BDNF" OR "KCC2" OR "estradiol" OR "testosterone" OR "epigenetics"). Equivalent strings, adapted to database-specific syntax, were applied in Scopus and Web of Science.

Eligibility prioritized studies establishing mechanistic, causal, or translational links between biological sex and the development or maintenance of CS. Three categories of evidence were considered: (i) preclinical investigations in rodent models that explicitly incorporated biological sex as an independent variable in the analysis of CS, neuropathic pain, or immune-mediated nociception; (ii) clinical and translational studies in human cohorts addressing sex-specific differences in pain perception, pharmacological treatment response, or hormonal biomarkers; and (iii) systematic reviews and meta-analyses providing theoretical frameworks for neuroimmune sexual dimorphism or evaluating sex-differential therapeutic outcomes. Articles published in English and Portuguese were eligible. Priority was accorded to peer-reviewed publications within the preceding decade; the foundational works of Tsuda et al. [9] and Coull et al. [10] were retained, given their status as the molecular cornerstone of the field. Conference abstracts, editorials without primary data, and non-peer-reviewed preprints were excluded. Study identification and selection were performed by the single author. Records retrieved from the three databases were screened by title and abstract for relevance to the review's analytical axes, and potentially eligible articles were assessed in full text against the eligibility criteria described above. As a single-author narrative synthesis, this process did not involve independent dual screening; this is acknowledged as a methodological limitation inherent to the narrative review format.

Eligible studies were qualitatively synthesized using a narrative approach structured around four analytical axes: (i) cellular identity of the primary pain-sustaining immune effector, (ii) molecular signalling cascade, (iii) hormonal modulation, and (iv) epigenetic consolidation. This framework was selected to serve the review’s primary clinical audience, interventional pain specialists, by translating molecular complexity into a mechanistically grounded therapeutic rationale. As a narrative synthesis, this review does not include a formal risk-of-bias assessment or quantitative meta-analysis; this limitation is acknowledged and discussed.

Beyond this qualitative synthesis, each mechanistic target in Table 1 was assigned an exploratory evidence grade to convey the translational maturity of the supporting data. This is an author-derived qualitative schema, not a validated quality-assessment instrument such as Grading of Recommendations Assessment, Development and Evaluation (GRADE), with three a priori tiers: Grade A, convergent preclinical and clinical evidence of sex-differential efficacy; Grade B, robust preclinical evidence with limited or emerging clinical data; and Grade C, preliminary mechanistic rationale only. It is intended as an interpretive aid, not a substitute for systematic quality appraisal, the absence of which is acknowledged as a limitation.

**Table 1:** Sex-stratified precision-analgesia framework BDNF: brain-derived neurotrophic factor; CNS: central nervous system; CSF1R: colony-stimulating factor 1 receptor; DNMT: DNA methyltransferase; DRG: dorsal root ganglion; DRGS: dorsal root ganglion stimulation; GPER: G-protein-coupled estrogen receptor; HDAC: histone deacetylase; HPG: hypothalamic-pituitary-gonadal; IFN-γ: interferon-γ; JAK: Janus kinase; KCC2: potassium-chloride cotransporter 2; miRNA: microRNA; Nav: voltage-gated sodium channel; NMDA: N-methyl-D-aspartate; P2X4R: P2X4 purinergic receptor; PAG: periaqueductal grey; RCT: randomized controlled trial; SCS: spinal cord stimulation; SNRI: serotonin-norepinephrine reuptake inhibitor; TNF-α: tumor necrosis factor-α; TrkB: tropomyosin receptor kinase B Mechanistic targets, drug classes, phenotypic applicability, evidence grading, and priority research questions. Evidence grade: A = convergent preclinical and clinical evidence of sex-differential efficacy; B = robust preclinical evidence with limited or emerging clinical data; C = preliminary mechanistic rationale only

Mechanistic target	Drug class/strategy	Primary sex phenotype	Evidence grade	Priority research question
P2X4R/microglial activation	P2X4 antagonists; CSF1R inhibitors; minocycline	Male	B	Sex-stratified efficacy in prospective RCTs with biological sex as a mandatory enrolment stratum; biomarker-driven patient selection
BDNF-TrkB-KCC2 axis	TrkB antagonists; KCC2 activators (e.g., CLP290); anti-BDNF monoclonal antibodies	Male	B	KCC2 restoration as a pharmacological strategy to re-establish GABAergic inhibition in male neuropathic pain; phase 1 dose-finding
T-cell/cytokine pathway (IFN-γ; TNF-α)	Anti-TNF biologics; IFN-γ neutralization; JAK inhibitors; Th17 modulators	Female	B-C	Repurposing of approved biologics (e.g., adalimumab, tofacitinib) in female-predominant chronic pain syndromes (fibromyalgia; chronic widespread pain)
NMDA receptor/estradiol-GPER axis	Low-dose ketamine, memantine, and selective GPER antagonists	Female (particularly perimenstrual/perimenopausal)	B	Menstrual-cycle-phase-informed ketamine dosing protocols; GPER-selective antagonists in phase 1 safety trials
Voltage-gated sodium channels (Nav1.7-1.9)	Selective Nav1.7/Nav1.8 blockers (e.g., suzetrigine); topical sodium channel antagonists	Female (estradiol-driven DRG upregulation)	B	Sex-stratified analysis of Nav blocker RCTs; serum estradiol as a predictive biomarker of Nav expression and analgesic response [21,30]
Hypothalamic-pituitary-gonadal (HPG) axis	Testosterone replacement therapy (hypogonadal patients); anti-estrogenic adjunct strategies	Both sexes (sex-differentiated dosing)	B-C	Systematic androgen deficiency screening in treatment-refractory chronic pain; hormonal biomarker panels as baseline eligibility criteria
Descending inhibitory control (PAG-spinal)	Spinal cord stimulation; dorsal root ganglion stimulation; SNRIs; sex-stratified opioid dosing	Both sexes (sex-differentiated efficacy)	A-B	Sex as a mandatory stratification variable in all SCS/DRGS outcome registries; development of sex-specific opioid dosing algorithms
Epigenetic maintenance (HDAC; DNMT; miRNA)	HDAC inhibitors; DNMT modulators; microRNA mimics/anti-miRs	Both sexes (sex-differentiated landscape)	C	Sex-specific CNS epigenetic biomarker discovery; phase 1 CNS safety profiling of epigenetic analgesic candidates

Beyond the neuron: the quadripartite neuroimmune model

The conceptual evolution of CS mirrors broader transformations in neuroscience. For much of the twentieth century, CS was understood in purely synaptic terms, as a form of long-term potentiation (LTP) in the dorsal horn driven by NMDA receptor-mediated calcium influx and downstream kinase activation [8]. Neurons were the protagonists; other cell types were relegated to supporting roles. This model was elegant, tractable, and incomplete. Contemporary neuroimmunology has replaced it with a multicellular model in which the synapse is embedded within a dynamic immune microenvironment. The conceptual progression from the “tripartite synapse,” in which astrocytes were recognized as active participants, to a quadripartite model incorporating microglia and infiltrating peripheral immune cells represents a substantial paradigm shift [22,23].

In the quadripartite model, activated microglia are not passive bystanders to neuronal pathology; they are among its architects. Following peripheral nerve injury, neurons emit molecular distress signals (adenosine triphosphate (ATP), fractalkine (CX3CL1), and colony-stimulating factor 1 (CSF1)) that function as chemotactic beacons, recruiting and activating spinal microglia [9,22,24]. The activated microglia, in turn, release neuroactive mediators that alter synaptic gain and inhibitory tone. The identity of these mediators, and, critically, of the cells that release them, depends fundamentally on the biological sex of the organism.

The male nociceptive program: the P2X4-BDNF-KCC2 cascade

In males, the cellular transition from acute to chronic pain is governed by a hierarchical, microglial-dependent signalling cascade now characterized at near-molecular resolution. The initiating event is the injury-induced transcriptional upregulation of P2X4 purinergic receptors (P2X4R) on the surface of spinal microglia, a phenomenon absent in uninjured animals and sex-restricted in its functional consequences [9,25]. Upon stimulation by extracellular ATP released from injured primary afferent terminals, P2X4R activation triggers calcium-dependent exocytotic release of brain-derived BDNF from microglia [9,10]. BDNF subsequently binds tropomyosin receptor kinase B (TrkB) on postsynaptic lamina I neurons, initiating a cascade that downregulates KCC2.

Under homeostatic conditions, KCC2 maintains a low intraneuronal Cl⁻ concentration, preserving the hyperpolarizing electrochemical gradient essential for inhibitory GABAergic and glycinergic neurotransmission. Loss of KCC2 inverts this gradient: intracellular Cl⁻ accumulates, and subsequent GABA receptor activation paradoxically drives Cl⁻ outward, depolarizing rather than hyperpolarizing the postsynaptic neuron [26]. This conversion of an inhibitory circuit into an excitatory one, disinhibition, constitutes the primary cellular mechanism of neuropathic mechanical allodynia in the male nervous system [10]. While the cascade itself is well-established, its prescribing corollary remains a mechanistic prediction rather than a validated clinical finding: when the chloride gradient has been pathologically reversed by microglial BDNF, pharmacologically enhancing GABAergic tone via gabapentinoids may, in principle, yield attenuated or even paradoxical effects [10,26]. This represents not a failure of pharmacology but a failure of mechanistic phenotyping prior to prescribing.

The female nociceptive program: the adaptive immune bypass

Among the most influential findings in pain neuroscience over the past decade is the demonstration by Sorge et al. that the P2X4-BDNF-KCC2 microglial axis, so central to pain chronification in males, is largely functionally dispensable in females [11,27]. Pharmacological blockade of microglial activation, genetic depletion of P2X4 receptors, and administration of minocycline reversed allodynia in male mice while producing little measurable benefit in females, despite equivalent microglial activation [11]. The microglia were present; they did not appear to drive the sustained sensitized state.

In the female spinal cord, the predominant effector cell of CS is the T-lymphocyte. Following nociceptive injury, a sex-specific infiltration of adaptive immune cells, predominantly of Th1 and Th17 lineage, is observed within the spinal cord parenchyma [6,11,28,29]. These cells elaborate a pro-inflammatory cytokine milieu rich in interferon-γ (IFN-γ) and tumor necrosis factor-α (TNF-α), which act directly on dorsal horn neurons to upregulate AMPA and NMDA receptor surface expression, reduce GABAergic inhibitory tone, and facilitate LTP [6,11]. This T-cell-mediated excitation largely bypasses the BDNF-KCC2 axis, rendering the female sensitization program structurally resistant to interventions targeting the microglial tier of the cascade [6,11]. This observation suggests a clinical corollary that, while not yet directly tested, is mechanistically coherent: trials of glial-targeted analgesics, designed and validated in male preclinical models, have overwhelmingly enrolled patients from female-predominant patient populations. This biological mismatch offers a plausible explanation for a pattern of clinical trial failure previously attributed to pharmacological inadequacy: the agents may act on the targeted mechanism in patients in whom that mechanism is operative, while failing in those in whom it is not.

Hormonal modulation: estradiol as pronociceptive amplifier, testosterone as neuroimmune brake

The cellular dichotomy described above does not operate in a hormonal vacuum. Whereas the preceding mechanisms operate predominantly within the spinal dorsal horn, hormonal modulation acts across the full neuraxis, encompassing peripheral nociceptors and the dorsal root ganglion (DRG), the spinal cord, and supraspinal structures such as the periaqueductal grey (PAG). The reader should note this widening of anatomical scope: the sex-steroid effects described below are not confined to the central compartment but extend to peripheral and descending circuits that together shape nociceptive gain. Gonadal steroids are pleiotropic regulators of the neuroimmune environment, continuously modulating synaptic excitability, glial reactivity, and immune-cell trafficking [13,14]. 17β-estradiol occupies a position of particular mechanistic prominence. Its pro-nociceptive actions are mediated through a dual architecture: a rapid nongenomic arm, operating through membrane-localized G-protein-coupled estrogen receptor (GPER), that acutely potentiates NMDA receptor currents, amplifies calcium influx, and lowers the threshold for activity-dependent LTP; and a slower genomic arm that transcriptionally upregulates nociceptor-enriched voltage-gated sodium channel isoforms Nav1.7, Nav1.8, and Nav1.9 in the DRG [13,30]. The former mechanism accounts for fluctuations in pain sensitivity across the menstrual cycle; the latter underlies the enduring peripheral hyperexcitability characteristic of many female-predominant chronic pain syndromes [30]. Together, these actions position estradiol as a biological gain amplifier, not merely modulating pain at the perceptual threshold but actively promoting the establishment and consolidation of CS.

Testosterone presents a mechanistically contrasting profile. Converging experimental and translational evidence indicates that testosterone attenuates nociceptive processing through at least three mechanisms: (i) direct suppression of microglial P2X4R expression and pro-inflammatory cytokine release, (ii) enhancement of descending inhibitory control through modulation of PAG opioidergic and serotonergic output, and (iii) facilitation of endogenous cannabinoid tone [14,15]. These antinociceptive effects have clinical correlates: hypogonadal men demonstrate elevated pain sensitivity and reduced conditioned pain modulation relative to eugonadal controls, while physiological testosterone variation in healthy women is inversely correlated with experimental pain responsiveness [15]. On this basis, androgen deficiency may represent an underrecognized contributor to treatment-refractory chronic pain in both sexes, a hypothesis that warrants prospective clinical evaluation. The estradiol-to-testosterone ratio can be conceptualized, within this framework, as a neuroimmune set point, a model whose clinical translation into endocrine-informed analgesic decision-making remains to be established [18].

Epigenetic consolidation: molecular architecture of pain memory

The third and deepest dimension of biological sex in chronic pain is epigenetic. If the neuroimmune dichotomy identifies which cells sustain CS, and gonadal hormones establish the excitability threshold at which it is triggered, epigenetic mechanisms explain why CS persists, often long after the precipitating injury has resolved and the hormonal environment has shifted. Epigenetic regulation constitutes the molecular memory of nociceptive experience. The principal mechanisms implicated-DNA methylation, posttranslational histone modification, chromatin remodelling, and noncoding RNA networks-share a common functional logic: they alter gene transcription without modifying the DNA sequence, enabling a transient biological event to produce durable phenotypic consequences. In the context of CS, these mechanisms sustain pro-excitatory ion channel expression, cytokine receptor surface density, and synaptic scaffolding protein availability in spinal cord and DRG neurons and glia, maintaining the sensitized state in the absence of ongoing peripheral input [16,17,31,32].

Sex-specific epigenetic programming adds a critical dimension. Gonadal hormones interact extensively with the epigenome: estrogen-responsive elements regulate the transcription of key nociceptive genes; androgen-receptor signalling modulates chromatin accessibility in pain-relevant tissues; and hormone-sensitive microRNAs post-transcriptionally regulate pro-inflammatory cytokine mRNA stability [13,16]. These interactions create sex-differentiated epigenetic landscapes that may help account for inter-individual variability in chronic pain development after comparable injuries. From a therapeutic standpoint, this dimension is conceptually transformative: it reframes chronic pain not as a permanent neurological deficit but as a transcriptionally maintained pathological state, and, in principle, a reversible one. Histone deacetylase (HDAC) inhibitors, DNA methyltransferase (DNMT) modulators, and microRNA-targeting strategies are under preclinical investigation as analgesic candidates [17], and their development requires sex-stratified experimental design from the outset.

Clinical and translational implications: toward sex-stratified precision analgesia

The mechanistic architecture described above converges on a clinical conclusion: the biological substrate of chronic pain differs between males and females in ways that have therapeutic consequences. The current analgesic landscape, built on sex-undifferentiated biology, is structurally misaligned with the disease it seeks to treat. Table 2 synthesizes the key mechanistic dichotomies and their clinical correlates. Table 1 maps these mechanisms onto a sex-stratified precision-analgesia framework with explicit evidence grading.

**Table 2:** Sex-specific neuroimmune mechanisms of central sensitization ATP: adenosine triphosphate; AMPA: α-amino-3-hydroxy-5-methyl-4-isoxazolepropionic acid; BDNF: brain-derived neurotrophic factor; DRG: dorsal root ganglion; GABA: γ-aminobutyric acid; GPER: G-protein-coupled estrogen receptor; IFN-γ: interferon-γ; KCC2: potassium-chloride cotransporter 2; Nav: voltage-gated sodium channel; NMDA: N-methyl-D-aspartate; P2X4R: P2X4 purinergic receptor; PAG: periaqueductal grey; TNF-α: tumor necrosis factor-α; TrkB: tropomyosin receptor kinase B Cellular dichotomy, hormonal modulation, epigenetic regulation, and clinical correlates of central sensitization in males and females. Male and female phenotypes denote the predominant, rather than mutually exclusive, neuroimmune mechanism in each sex; overlap and context-dependent plasticity between pathways are discussed in the main text

Feature	Male phenotype	Female phenotype	Refs.	Clinical implication
Primary effector cell	Spinal microglia (activated, pro-inflammatory state)	Spinal T-lymphocytes (Th1/Th17 lineage)	[6,9-11]	Microglial inhibitors (minocycline; P2X4 antagonists) appear sex-selective, with limited benefit in female cohorts despite equivalent microglial activation
Initiating molecular signal	Peripheral nerve injury → ATP release → P2X4R upregulation on microglia	Nociceptive insult → sex-specific T-lymphocyte spinal infiltration	[9,11,28]	P2X4R antagonism represents a male-specific target; no analogous validated upstream female target is yet established
Key neuroimmune mediator	Microglial-derived BDNF	T-cell-derived IFN-γ and TNF-α	[6,10,11]	Anti-BDNF strategies may preferentially benefit males; TNF-α/IFN-γ blockade may preferentially benefit females
Mechanism of disinhibition	BDNF → TrkB → KCC2 downregulation → Cl⁻ gradient reversal → paradoxical GABAergic depolarization	Direct cytokine-mediated upregulation of AMPA/NMDA receptor surface density; KCC2 axis less central	[10,11]	Gabapentinoid efficacy may be attenuated in males with KCC2 dysregulation; cytokine blockade bypasses this mechanism
Hormonal modulator	Testosterone: suppresses P2X4R expression; facilitates PAG-mediated descending inhibition	17β-estradiol: potentiates NMDA currents (nongenomic, via GPER); upregulates Nav1.7–1.9 in DRG (genomic)	[13,15,30]	Androgen deficiency amplifies male nociceptive vulnerability; estrogen cycling underpins female pain fluctuation across the reproductive lifespan
Epigenetic contribution	Androgen-sensitive chromatin remodelling; androgen receptor-regulated microRNA networks	Estrogen-responsive element methylation; sex-differentiated DNA methylation of nociceptive loci	[16,17]	Epigenetic therapies (HDAC inhibitors; DNMT modulators) require sex-stratified preclinical development and dosing optimization
Response to microglial inhibition	Reversal of mechanical allodynia (robust in preclinical models; emerging in clinical investigation)	Limited measurable benefit; allodynia maintained via adaptive immune bypass	[6,11]	Sex should be a mandatory stratification variable, not a post hoc subgroup analysis, in all clinical trials of glial-targeted analgesics

In male-predominant phenotypes characterized by microglial dominance and KCC2 dysregulation, glial-targeted agents such as P2X4 antagonists, CSF1R inhibitors, and microglial-selective pharmacology represent mechanistically coherent interventional targets. In female-predominant phenotypes driven by adaptive immune signalling, anti-cytokine strategies such as TNF-α blockade, IFN-γ neutralization, and Th1/Th17-targeting immunomodulatory agents may offer a more biologically congruent approach; a precedent exists in inflammatory arthritis, where sex-differential responses to biological disease-modifying agents are increasingly recognized and informing prescribing practice [6,18]. Opioid therapy warrants specific reconsideration: sex differences in μ-opioid receptor density, endogenous opioid tone, and opioid-induced hyperalgesia are well-documented and consistent with the broader model advanced here [14]. The recent approval (January 2025) of suzetrigine, the first-in-class selective Nav1.8 inhibitor for acute pain, illustrates the clinical traction of ion-channel-targeted analgesia and underscores the need for sex-stratified efficacy analyses in registrational and postmarketing studies, given the sex-dimorphic expression of Nav1.7-1.9 [21]. For the interventional pain specialist, precision analgesia begins with biological stratification at the point of patient assessment: biological sex, endocrine status, and, where feasible, immunological phenotyping should be considered core clinical variables alongside pain intensity, duration, and distribution. The analgesic armamentarium is not lacking. What has been lacking is the biological precision required to deploy it correctly.

Discussion

This review advances the position that CS is not a pathophysiological monolith but a spectrum of mechanistically distinct neuroimmune states whose cellular determinants, hormonal dependencies, and epigenetic trajectories are shaped, at the most fundamental level, by biological sex. The evidence is now sufficiently mature to move beyond hypothesis: distinct immune cells sustain allodynia in male and female animals [6,11,28]; distinct receptor systems mediate hormonal sensitization [13,30]; and distinct transcriptional programs underlie epigenetic persistence [16,17]. The field has progressed from the observation that sex differences in pain exist to a mechanistic understanding of how and why they exist, and this transition demands a corresponding evolution in clinical practice.

The translational asymmetry at the heart of this review merits particular emphasis. The P2X4-BDNF-KCC2 cascade is among the most rigorously characterized pathways in pain neuroscience, the subject of landmark publications and the culmination of decades of microglial biology [9,10]. Yet it was derived almost exclusively from male animals, and its therapeutic translation has been correspondingly male-biased. The consistent failure of microglial inhibitors in female-predominant clinical cohorts has, until recently, been interpreted as evidence of pharmacological inadequacy. The mechanistic reframing offered here suggests an alternative interpretation: these are targeted therapies tested in the wrong target population [6]. This distinction is not semantic; it is the difference between abandoning a valid mechanism and redirecting it appropriately.

Nuancing the dichotomy: overlap, plasticity, and convergence

The microglia-versus-T-cell framework should be read as describing the predominant effector mechanism in each sex, not mutually exclusive pathways. Microglial activation occurs in both sexes after nerve injury; the sex difference lies in whether it is required to sustain hypersensitivity, not in its presence [11]. The dependence also appears context-dependent rather than hardwired, suggesting alternative routes to a shared endpoint rather than immutable, sex-locked circuits [6,11]. Moreover, irrespective of the upstream effector cell, the downstream consequences-dorsal-horn hyperexcitability, loss of inhibitory tone, and facilitated LTP-converge on a common final pathway. The divergence is thus most pronounced at the initiating immune effector and attenuates downstream. Rather than weakening the central thesis, this refines it: biological sex modulates which neuroimmune route predominates within a partially shared architecture. The clinical implication is unchanged, but this framing guards against assuming any individual patient conforms rigidly to a sex-typical mechanism.

Limitations of the current evidence base

Several limitations qualify the current state of knowledge. First, the majority of mechanistic data derive from rodent models; the degree to which the microglial/T-cell dichotomy is conserved in human spinal cord biology remains an open question. Human dorsal horn immunohistochemistry, spinal cord single-cell RNA sequencing, and cerebrospinal fluid cytokine profiling in sex-stratified chronic pain cohorts represent the most direct routes to closing this gap [6,18]. Second, the distinction between biological sex and gender, encompassing psychosocial, cultural, and experiential dimensions of pain, is rarely operationalized with sufficient rigor in clinical studies, introducing potential confounders into translational inference [18]. Third, the hormonal epigenetic landscape across the lifespan remains incompletely mapped, and this synthesis emphasizes short-term cyclic variation at the expense of major, durable endocrine transitions. The menopausal decline in 17β-estradiol and the age-related fall in testosterone represent permanent recalibrations of the neuroimmune set point rather than transient fluctuations and may help explain the altered prevalence, character, and treatment responsiveness of chronic pain in older demographics [33]. How these enduring shifts reshape the neuroimmune baseline, and whether they alter the predominant sensitization mechanism itself, is an important priority for research and endocrine-informed analgesic practice. Fourth, while epigenetic targets represent a compelling therapeutic frontier, the technical and safety challenges of CNS-directed epigenetic pharmacology remain substantial [16,17]. Fifth, as a narrative review, this synthesis does not include formal, instrument-based quality appraisal or quantitative meta-analysis; the evidence grades presented in Table 1 represent an exploratory, author-derived schema rather than a validated grading system, and future systematic reviews applying instruments such as GRADE will be needed to refine the evidence stratification proposed here. Additionally, the single-author design precludes the independent dual study selection characteristic of systematic reviews, and the search, while structured and reproducible in its sources and terms, was not registered a priori.

Clinical and research priorities

The research agenda these limitations define is concrete and actionable: (i) human spinal cord neuroimmune phenotyping in sex-stratified chronic pain cohorts, (ii) prospective registration of biological sex as a mandatory stratification variable in interventional pain trials, including neuromodulation registries [19], (iii) development of clinically deployable hormonal and immunological biomarkers to guide therapeutic selection, (iv) reanalysis of legacy clinical trial data with sex as a primary effect modifier, and (v) regulatory dialogue on sex-stratified efficacy reporting in analgesic drug approval, building on the recent precedent of suzetrigine and other emerging targeted analgesics [21]. The question is no longer whether sex is a relevant biological variable in pain medicine; it demonstrably is. The question is how to operationalize that knowledge in clinical trial design, biomarker development, and targeted therapeutic translation.

## Conclusions

Central sensitization is not a single, universal phenomenon but a sex-dimorphic neuroimmune process whose cellular architects, molecular signals, hormonal gatekeepers, and epigenetic consolidators appear to differ in fundamental respects between males and females. In males, the P2X4-BDNF-KCC2 microglial cascade drives disinhibition and allodynia, whereas in females, T-lymphocyte infiltration and direct cytokine-mediated excitation of dorsal horn neurons sustain chronic pain through a parallel circuit largely refractory to microglial-targeted intervention, with gonadal steroids and epigenetic mechanisms further shaping and consolidating each program. Much of this architecture has so far been delineated in preclinical models and still awaits full confirmation in humans, yet the convergence of the evidence is difficult to ignore: these differences are unlikely to be merely theoretical, given that the majority of chronic pain patients are women and that analgesic strategy has too often been built on a predominantly male biological substrate. Precision analgesia, therefore, calls for treatment that is stratified by sex, informed by endocrine context, and, where feasible, grounded in the neuroimmune phenotype of the individual patient. The molecular architecture of sex differences in pain is coming into increasingly sharp focus, and although much work remains, above all, adequately powered, sex-stratified clinical trials and validated biomarkers, incorporating biological sex as a primary variable in pain research and clinical care, are both sound science and, ultimately, a path toward more effective and equitable therapy for those who need it most.
